# Lanthanum ruthenium indide, La_21_Ru_9+*x*_In_5-*x*_ (*x* = 1.2)

**DOI:** 10.1107/S1600536810014509

**Published:** 2010-04-24

**Authors:** Anna I. Tursina, Sergei G. Cherviakov, Henri Noël, Vladimir V. Chernyshev, Yuri D. Seropegin

**Affiliations:** a Department of Chemistry, Moscow State University, Leninskie Gory 1/3, 119 992 Moscow, Russian Federation; b Laboratoire de Chimie du Solide et Matériaux, UMR6226 CNRS-Université de Rennes 1, Avenue du Général Leclerc, 30542 Rennes, France

## Abstract

La_21_Ru_9+*x*_In_5-*x*_ (Pearson symbol *tI*140) is isotypic to the filled Y_3_Rh_2_-type structure, from which it can be derived through an ordered substitution at two sites. One of the square-prismatic sites (site symmetry ..*m*) is occupied by a mixture of Ru and In atoms and one of the square-antiprismatic sites (4/*m*..) is fully occupied by In atoms.

## Related literature

For related structures, see: Zaremba *et al.* (2007 ▶); Moreau *et al.* (1976 ▶). For standardization of crystal structures, see: Gelato & Parthé (1987 ▶).

## Experimental

### 

#### Crystal data

La_21_Ru_10.16_In_3.84_
*M* *_r_* = 4384.89
Tetragonal, 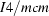

*a* = 12.1298 (3) Å
*c* = 25.9820 (7) Å
*V* = 3822.79 (17) Å^3^
*Z* = 4
Mo *K*α radiation
μ = 28.98 mm^−1^
*T* = 293 K
0.06 × 0.05 × 0.05 mm

#### Data collection

Nonius KappaCCD diffractometer
Absorption correction: for a sphere (*WinGX*; Farrugia, 1999 ▶) *T*_min_ = 0.243, *T*_max_ = 0.261
22423 measured reflections
1202 independent reflections
927 reflections with *I* > 2σ(*I*)
*R*_int_ = 0.087

#### Refinement

*R*[*F* ^2^ > 2σ(*F* ^2^)] = 0.036
*wR*(*F* ^2^) = 0.062
*S* = 1.12
1202 reflections
53 parameters
Δρ_max_ = 2.00 e Å^−3^
Δρ_min_ = −2.74 e Å^−3^

### 

Data collection: *COLLECT* (Nonius, 1998 ▶); cell refinement: *DENZO* (Otwinowski & Minor, 1997 ▶); data reduction: *DENZO*; program(s) used to solve structure: *SHELXS97* (Sheldrick, 2008 ▶); program(s) used to refine structure: *SHELXL97* (Sheldrick, 2008 ▶); molecular graphics: *DIAMOND* (Brandenburg, 1999 ▶); software used to prepare material for publication: *SHELXL97*.

## Supplementary Material

Crystal structure: contains datablocks I, global. DOI: 10.1107/S1600536810014509/mg2098sup1.cif

Structure factors: contains datablocks I. DOI: 10.1107/S1600536810014509/mg2098Isup2.hkl

Additional supplementary materials:  crystallographic information; 3D view; checkCIF report

## Figures and Tables

**Fig. 1.**
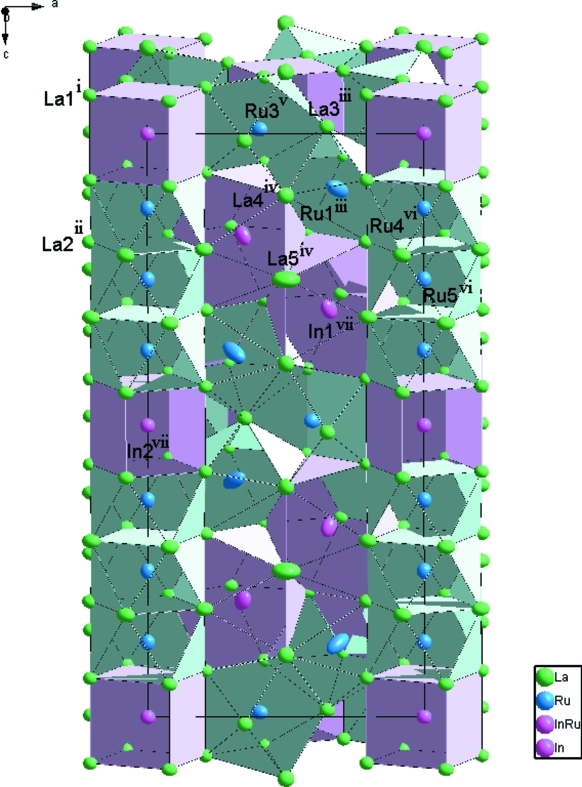
Structure of the title compound emphasizing the coordination polyhedra, with atom labelling shown and displacement ellipsoids drawn at the 50% probability level.

## supplementary crystallographic information

### Comment

New rare-earth metal-rich indium compounds RE_3_T_2-__x_In_x_ (RE = Gd, Tb, Dy, Ho, Er, Tm; T = Rh, Pd, Ir) have been recently synthesized (Zaremba et al., 2007). They can be regarded as extensions of the parent binaries RE_3_T_2_ with either the Y_3_Rh_2_- (T = Rh, Ir) or U_3_Si_2_-type (T = Pd) structures into the ternary RE–T–In systems. In contrast, La_21_Ru_9+__x_In_5-__x_, presented here, is strictly a ternary compound with no corresponding La-Ru binary of the same stoichiometry.

In the Y_3_Rh_2_–type structure, six crystallographically independent transition metal sites are available with trigonal prismatic, square prismatic, and square antiprismatic coordination environments (Moreau et al., 1976). The structure of La_21_Ru_9+__x_In_5-__x_ is derived through an ordered substitution at two sites, with the square prismatic site (16l) occupied by a mixture of Ru and In atoms and one of the square antiprismatic sites (4c) occupied fully by In atoms (Fig. 1). This suggests the existence of a solid solution, as confirmed by EDX measurements which revealed a homogeneity range of ca. 3 at.% in La_21_Ru_9+__x_In_5-__x_.

### Experimental

The title compound was prepared by arc-melting of the constituent elements (La, 99.8%; Ru, 99.9%, In, 99.999%) under a high purity argon atmosphere on a water-cooled cooper hearth. The arc-melted button, with nominal composition La_59.26_Ru_29.63_In_11.11_, was turned over and remelted to ensure its homogeneity. The weight loss was less than 1%. The sample was annealed in an evacuated quartz ampoule at 870 K for 600 h and quenched in cold water. The single crystal was selected from the crushed sample.

EDX analysis of the majority phase in a number of samples revealed that the composition of the new compound ranges from La_58.8_Ru_26.2_In_15.0_ to La_61.1_Ru_28.3_In_10.7_ with an uncertainty of about 1 at.% for each element. Thus the homogeneity range of the title compound is approximately 3 at.% at 870 K.

### Refinement

The atomic parameters were standardized with the program STRUCTURE TIDY (Gelato & Parthé, 1987). The highest peak and the deepest hole in the final difference map are located 0.69 Å from La2 and 0.82 Å, respectively, from Ru1.

### Crystal data

**Table:**

La_21_Ru_10.16_In_3.84_	*D*_x_ = 7.619 Mg m^−^^3^
*M**_r_* = 4384.89	Mo *K*α radiation, λ = 0.71073 Å
Tetragonal, *I*4/*m**c**m*	Cell parameters from 12585 reflections
Hall symbol: -I 4 2c	θ = 2.9–27.5°
*a* = 12.1298 (3) Å	µ = 28.98 mm^−^^1^
*c* = 25.9820 (7) Å	*T* = 293 K
*V* = 3822.79 (17) Å^3^	Prism, metallic-dark-grey
*Z* = 4	0.06 × 0.05 × 0.05 mm
*F*(000) = 7329	

### Data collection

**Table:**

Nonius KappaCCD diffractometer	1202 independent reflections
Radiation source: fine-focus sealed tube	927 reflections with *I* > 2σ(*I*)
graphite	*R*_int_ = 0.087
φ and ω scans	θ_max_ = 27.5°, θ_min_ = 3.7°
Absorption correction: for a sphere (*WinGX*; Farrugia, 1999)	*h* = −15→15
*T*_min_ = 0.243, *T*_max_ = 0.261	*k* = −15→15
22423 measured reflections	*l* = −33→32

### Refinement

**Table:**

Refinement on *F*^2^	0 restraints
Least-squares matrix: full	Primary atom site location: structure-invariant direct methods
*R*[*F*^2^ > 2σ(*F*^2^)] = 0.036	Secondary atom site location: difference Fourier map
*wR*(*F*^2^) = 0.062	*w* = 1/[σ^2^(*F*_o_^2^) + (0.0131*P*)^2^ + 224.3566*P*] where *P* = (*F*_o_^2^ + 2*F*_c_^2^)/3
*S* = 1.12	(Δ/σ)_max_ < 0.001
1202 reflections	Δρ_max_ = 2.00 e Å^−^^3^
53 parameters	Δρ_min_ = −2.74 e Å^−^^3^

### Special details

**Table:**

Geometry. All esds (except the esd in the dihedral angle between two l.s. planes) are estimated using the full covariance matrix. The cell esds are taken into account individually in the estimation of esds in distances, angles and torsion angles; correlations between esds in cell parameters are only used when they are defined by crystal symmetry. An approximate (isotropic) treatment of cell esds is used for estimating esds involving l.s. planes.
Refinement. Refinement of *F*^2^ against ALL reflections. The weighted *R*-factor wR and goodness of fit *S* are based on *F*^2^, conventional *R*-factors *R* are based on *F*, with *F* set to zero for negative *F*^2^. The threshold expression of *F*^2^ > σ(*F*^2^) is used only for calculating *R*-factors(gt) etc. and is not relevant to the choice of reflections for refinement. *R*-factors based on *F*^2^ are statistically about twice as large as those based on *F*, and *R*- factors based on ALL data will be even larger.

### Fractional atomic coordinates and isotropic or equivalent isotropic displacement parameters (Å^2^)

**Table:**

	*x*	*y*	*z*	*U*_iso_*/*U*_eq_	Occ. (<1)
La1	0.07818 (5)	0.20842 (5)	0.07297 (2)	0.01886 (15)	
La2	0.20391 (6)	0.07950 (6)	0.19170 (2)	0.02653 (17)	
La3	0.85106 (8)	0.35106 (8)	0.0000	0.0208 (3)	
La4	0.0000	0.5000	0.10584 (5)	0.0255 (3)	
La5	0.0000	0.5000	0.2500	0.0615 (8)	
Ru1	0.81308 (11)	0.31308 (11)	0.10986 (6)	0.0429 (4)	
Ru2	0.65628 (8)	0.15628 (8)	0.18661 (5)	0.0287 (4)	0.29 (4)
Ru3	0.59671 (12)	0.09671 (12)	0.0000	0.0247 (4)	
Ru4	0.0000	0.0000	0.12798 (6)	0.0207 (4)	
Ru5	0.0000	0.0000	0.2500	0.0213 (5)	
In1	0.65628 (8)	0.15628 (8)	0.18661 (5)	0.0287 (4)	0.71 (4)
In2	0.0000	0.0000	0.0000	0.0200 (5)	

### Atomic displacement parameters (Å^2^)

**Table:**

	*U*^11^	*U*^22^	*U*^33^	*U*^12^	*U*^13^	*U*^23^
La1	0.0226 (3)	0.0185 (3)	0.0154 (3)	−0.0006 (3)	0.0001 (2)	0.0002 (2)
La2	0.0305 (4)	0.0311 (4)	0.0180 (3)	−0.0036 (3)	0.0003 (3)	−0.0001 (3)
La3	0.0218 (4)	0.0218 (4)	0.0188 (6)	0.0021 (6)	0.000	0.000
La4	0.0260 (4)	0.0260 (4)	0.0247 (7)	0.0035 (6)	0.000	0.000
La5	0.0793 (13)	0.0793 (13)	0.0258 (12)	0.000	0.000	0.000
Ru1	0.0471 (6)	0.0471 (6)	0.0345 (8)	0.0213 (8)	−0.0168 (6)	−0.0168 (6)
Ru2	0.0244 (5)	0.0244 (5)	0.0372 (8)	0.0073 (5)	−0.0070 (4)	−0.0070 (4)
Ru3	0.0270 (6)	0.0270 (6)	0.0202 (9)	0.0055 (8)	0.000	0.000
Ru4	0.0195 (5)	0.0195 (5)	0.0232 (9)	0.000	0.000	0.000
Ru5	0.0199 (8)	0.0199 (8)	0.0241 (12)	0.000	0.000	0.000
In1	0.0244 (5)	0.0244 (5)	0.0372 (8)	0.0073 (5)	−0.0070 (4)	−0.0070 (4)
In2	0.0206 (7)	0.0206 (7)	0.0190 (10)	0.000	0.000	0.000

### Geometric parameters (Å, °)

**Table:**

La1—Ru1^i^	3.0174 (15)	La4—La2^i^	4.3365 (10)
La1—Ru3^ii^	3.0385 (12)	La5—In1^xxix^	3.1464 (14)
La1—Ru4	3.0550 (10)	La5—Ru2^xxix^	3.1464 (14)
La1—In2	3.2992 (6)	La5—In1^xii^	3.1464 (14)
La1—In1^i^	3.5084 (13)	La5—Ru2^xii^	3.1464 (14)
La1—Ru2^i^	3.5084 (13)	La5—In1^i^	3.1464 (14)
La1—Ru1^iii^	3.5875 (11)	La5—In1^xxv^	3.1464 (14)
La1—La2^iv^	3.6299 (9)	La5—Ru2^i^	3.1464 (14)
La1—La1^v^	3.6607 (13)	La5—Ru2^xxv^	3.1464 (14)
La1—La4	3.7600 (7)	La5—La4^xxx^	3.7457 (13)
La1—La3^iii^	3.7653 (7)	La5—La2^i^	4.0154 (7)
La1—La2	3.7798 (9)	La5—La2^iv^	4.0154 (7)
La1—La1^vi^	3.7919 (11)	La5—La2^xxxi^	4.0154 (7)
La1—La1^iv^	3.8185 (9)	La5—La2^xii^	4.0154 (7)
La1—La1^vii^	3.8185 (9)	La5—La2^xxviii^	4.0154 (7)
La1—La3^ii^	3.8822 (12)	La5—La2^xxv^	4.0154 (7)
La2—Ru1^i^	2.8235 (14)	La5—La2^xxix^	4.0154 (7)
La2—Ru5	3.0565 (7)	La5—La2^x^	4.0154 (7)
La2—Ru4	3.1286 (11)	Ru1—La2^xxiii^	2.8235 (14)
La2—In1^i^	3.2592 (13)	Ru1—La2^xxii^	2.8235 (14)
La2—Ru2^i^	3.2592 (13)	Ru1—La1^xxii^	3.0174 (15)
La2—In1^viii^	3.3276 (8)	Ru1—La1^xxiii^	3.0174 (15)
La2—Ru2^viii^	3.3276 (8)	Ru1—La4^xvi^	3.2082 (19)
La2—La2^ix^	3.5915 (13)	Ru1—La1^xvi^	3.5875 (11)
La2—La1^vii^	3.6299 (9)	Ru1—La1^xx^	3.5875 (11)
La2—La2^x^	3.7060 (13)	Ru2—La5^xii^	3.1464 (14)
La2—In1^xi^	3.7068 (12)	Ru2—La2^xxii^	3.2592 (13)
La2—Ru2^xi^	3.7068 (12)	Ru2—La2^xxiii^	3.2592 (13)
La2—La2^v^	3.7154 (14)	Ru2—La2^v^	3.3276 (8)
La2—La2^vii^	3.7544 (10)	Ru2—La2^viii^	3.3276 (8)
La2—La2^iv^	3.7544 (10)	Ru2—La4^xiii^	3.4047 (15)
La2—La5^xii^	4.0154 (7)	Ru2—La1^xxii^	3.5084 (13)
La2—La4^xiii^	4.3365 (10)	Ru2—La1^xxiii^	3.5084 (13)
La3—Ru1^vi^	2.9278 (17)	Ru2—La2^xxxii^	3.7068 (12)
La3—Ru1	2.9278 (17)	Ru2—La2^xi^	3.7068 (12)
La3—Ru3^xiv^	3.0463 (16)	Ru3—La1^xiv^	3.0385 (12)
La3—Ru3^xv^	3.0463 (16)	Ru3—La1^xxii^	3.0385 (12)
La3—La4^xvi^	3.7536 (13)	Ru3—La1^xxiii^	3.0385 (12)
La3—La4^xvii^	3.7536 (13)	Ru3—La1^xxi^	3.0385 (12)
La3—La1^xvi^	3.7653 (7)	Ru3—La3^ii^	3.0463 (16)
La3—La1^xviii^	3.7653 (7)	Ru3—La3^xxxiii^	3.0463 (16)
La3—La1^xix^	3.7653 (7)	Ru3—La4^xiv^	3.2115 (15)
La3—La1^xx^	3.7653 (7)	Ru3—La4^xiii^	3.2115 (15)
La3—La1^xiv^	3.8822 (12)	Ru4—La1^xxxiv^	3.0550 (10)
La3—La1^xxi^	3.8822 (12)	Ru4—La1^iv^	3.0550 (10)
La3—La1^xxii^	3.8822 (12)	Ru4—La1^vii^	3.0550 (10)
La3—La1^xxiii^	3.8822 (12)	Ru4—La2^iv^	3.1286 (11)
La4—Ru1^xxiv^	3.2082 (19)	Ru4—La2^xxxiv^	3.1286 (11)
La4—Ru1^iii^	3.2082 (19)	Ru4—La2^vii^	3.1286 (11)
La4—Ru3^xxv^	3.2115 (15)	Ru5—La2^x^	3.0565 (7)
La4—Ru3^ii^	3.2115 (15)	Ru5—La2^xxxiv^	3.0565 (7)
La4—In1^i^	3.4047 (15)	Ru5—La2^vii^	3.0565 (7)
La4—In1^xxv^	3.4047 (15)	Ru5—La2^ix^	3.0565 (7)
La4—Ru2^i^	3.4047 (15)	Ru5—La2^xxxv^	3.0565 (7)
La4—Ru2^xxv^	3.4047 (15)	Ru5—La2^xxxvi^	3.0565 (7)
La4—La5	3.7457 (13)	Ru5—La2^iv^	3.0565 (7)
La4—La3^iii^	3.7536 (13)	In2—La1^xxxvii^	3.2992 (6)
La4—La3^xvii^	3.7536 (13)	In2—La1^iv^	3.2992 (6)
La4—La1^v^	3.7600 (7)	In2—La1^xxxiv^	3.2992 (6)
La4—La1^xxvi^	3.7600 (7)	In2—La1^vii^	3.2992 (6)
La4—La1^xxvii^	3.7600 (7)	In2—La1^xxxviii^	3.2992 (6)
La4—La2^xxviii^	4.3365 (10)	In2—La1^vi^	3.2992 (6)
La4—La2^xxv^	4.3365 (10)	In2—La1^xxxix^	3.2992 (6)
La4—La2^iv^	4.3365 (10)		
			
Ru1^i^—La1—Ru3^ii^	101.28 (4)	Ru2^xxv^—La4—La1^v^	143.53 (3)
Ru1^i^—La1—Ru4	94.19 (3)	La5—La4—La1^v^	103.13 (2)
Ru3^ii^—La1—Ru4	163.46 (3)	La3^iii^—La4—La1^v^	99.50 (3)
Ru1^i^—La1—In2	112.81 (4)	La3^xvii^—La4—La1^v^	60.151 (15)
Ru3^ii^—La1—In2	104.998 (19)	La1—La4—La1^v^	58.26 (2)
Ru4—La1—In2	62.97 (3)	Ru1^xxiv^—La4—La1^xxvi^	119.600 (14)
Ru1^i^—La1—In1^i^	61.19 (4)	Ru1^iii^—La4—La1^xxvi^	61.370 (13)
Ru3^ii^—La1—In1^i^	98.12 (2)	Ru3^xxv^—La4—La1^xxvi^	50.94 (3)
Ru4—La1—In1^i^	94.43 (3)	Ru3^ii^—La4—La1^xxvi^	103.96 (4)
In2—La1—In1^i^	156.88 (2)	In1^i^—La4—La1^xxvi^	143.53 (3)
Ru1^i^—La1—Ru2^i^	61.19 (4)	In1^xxv^—La4—La1^xxvi^	58.39 (2)
Ru3^ii^—La1—Ru2^i^	98.12 (2)	Ru2^i^—La4—La1^xxvi^	143.53 (3)
Ru4—La1—Ru2^i^	94.43 (3)	Ru2^xxv^—La4—La1^xxvi^	58.39 (2)
In2—La1—Ru2^i^	156.88 (2)	La5—La4—La1^xxvi^	103.13 (2)
In1^i^—La1—Ru2^i^	0.00 (2)	La3^iii^—La4—La1^xxvi^	60.151 (15)
Ru1^i^—La1—Ru1^iii^	142.38 (5)	La3^xvii^—La4—La1^xxvi^	99.50 (3)
Ru3^ii^—La1—Ru1^iii^	87.58 (5)	La1—La4—La1^xxvi^	115.02 (2)
Ru4—La1—Ru1^iii^	83.66 (3)	La1^v^—La4—La1^xxvi^	153.75 (4)
In2—La1—Ru1^iii^	99.62 (3)	Ru1^xxiv^—La4—La1^xxvii^	61.370 (13)
In1^i^—La1—Ru1^iii^	81.47 (3)	Ru1^iii^—La4—La1^xxvii^	119.600 (14)
Ru2^i^—La1—Ru1^iii^	81.47 (3)	Ru3^xxv^—La4—La1^xxvii^	50.94 (3)
Ru1^i^—La1—La2^iv^	103.08 (4)	Ru3^ii^—La4—La1^xxvii^	103.96 (4)
Ru3^ii^—La1—La2^iv^	125.52 (4)	In1^i^—La4—La1^xxvii^	143.53 (3)
Ru4—La1—La2^iv^	55.00 (3)	In1^xxv^—La4—La1^xxvii^	58.39 (2)
In2—La1—La2^iv^	108.99 (2)	Ru2^i^—La4—La1^xxvii^	143.53 (3)
In1^i^—La1—La2^iv^	55.539 (19)	Ru2^xxv^—La4—La1^xxvii^	58.39 (2)
Ru2^i^—La1—La2^iv^	55.539 (19)	La5—La4—La1^xxvii^	103.13 (2)
Ru1^iii^—La1—La2^iv^	46.06 (3)	La3^iii^—La4—La1^xxvii^	99.50 (3)
Ru1^i^—La1—La1^v^	52.66 (2)	La3^xvii^—La4—La1^xxvii^	60.151 (15)
Ru3^ii^—La1—La1^v^	52.959 (19)	La1—La4—La1^xxvii^	153.75 (4)
Ru4—La1—La1^v^	143.57 (2)	La1^v^—La4—La1^xxvii^	115.02 (2)
In2—La1—La1^v^	138.165 (11)	La1^xxvi^—La4—La1^xxvii^	58.26 (2)
In1^i^—La1—La1^v^	58.553 (15)	Ru1^xxiv^—La4—La2^xxviii^	40.60 (3)
Ru2^i^—La1—La1^v^	58.553 (15)	Ru1^iii^—La4—La2^xxviii^	136.53 (4)
Ru1^iii^—La1—La1^v^	112.56 (3)	Ru3^xxv^—La4—La2^xxviii^	102.66 (2)
La2^iv^—La1—La1^v^	112.533 (15)	Ru3^ii^—La4—La2^xxviii^	131.435 (17)
Ru1^i^—La1—La4	104.32 (3)	In1^i^—La4—La2^xxviii^	91.16 (3)
Ru3^ii^—La1—La4	55.15 (3)	In1^xxv^—La4—La2^xxviii^	49.124 (14)
Ru4—La1—La4	126.43 (3)	Ru2^i^—La4—La2^xxviii^	91.16 (3)
In2—La1—La4	141.15 (2)	Ru2^xxv^—La4—La2^xxviii^	49.124 (14)
In1^i^—La1—La4	55.73 (3)	La5—La4—La2^xxviii^	59.041 (17)
Ru2^i^—La1—La4	55.73 (3)	La3^iii^—La4—La2^xxviii^	151.947 (19)
Ru1^iii^—La1—La4	51.71 (3)	La3^xvii^—La4—La2^xxviii^	82.601 (16)
La2^iv^—La1—La4	71.84 (2)	La1—La4—La2^xxviii^	147.12 (2)
La1^v^—La1—La4	60.869 (10)	La1^v^—La4—La2^xxviii^	96.692 (16)
Ru1^i^—La1—La3^iii^	152.94 (3)	La1^xxvi^—La4—La2^xxviii^	96.726 (16)
Ru3^ii^—La1—La3^iii^	51.86 (3)	La1^xxvii^—La4—La2^xxviii^	52.689 (13)
Ru4—La1—La3^iii^	112.87 (3)	Ru1^xxiv^—La4—La2^xxv^	40.60 (3)
In2—La1—La3^iii^	81.52 (2)	Ru1^iii^—La4—La2^xxv^	136.53 (4)
In1^i^—La1—La3^iii^	113.98 (3)	Ru3^xxv^—La4—La2^xxv^	131.435 (17)
Ru2^i^—La1—La3^iii^	113.98 (3)	Ru3^ii^—La4—La2^xxv^	102.66 (2)
Ru1^iii^—La1—La3^iii^	46.85 (3)	In1^i^—La4—La2^xxv^	49.124 (14)
La2^iv^—La1—La3^iii^	92.826 (19)	In1^xxv^—La4—La2^xxv^	91.16 (3)
La1^v^—La1—La3^iii^	101.09 (2)	Ru2^i^—La4—La2^xxv^	49.124 (14)
La4—La1—La3^iii^	59.84 (2)	Ru2^xxv^—La4—La2^xxv^	91.16 (3)
Ru1^i^—La1—La2	47.47 (3)	La5—La4—La2^xxv^	59.041 (17)
Ru3^ii^—La1—La2	143.25 (3)	La3^iii^—La4—La2^xxv^	151.947 (19)
Ru4—La1—La2	53.21 (3)	La3^xvii^—La4—La2^xxv^	82.601 (16)
In2—La1—La2	105.542 (19)	La1—La4—La2^xxv^	96.726 (16)
In1^i^—La1—La2	52.97 (2)	La1^v^—La4—La2^xxv^	52.689 (13)
Ru2^i^—La1—La2	52.97 (2)	La1^xxvi^—La4—La2^xxv^	147.12 (2)
Ru1^iii^—La1—La2	106.86 (3)	La1^xxvii^—La4—La2^xxv^	96.692 (16)
La2^iv^—La1—La2	60.85 (2)	La2^xxviii^—La4—La2^xxv^	50.73 (2)
La1^v^—La1—La2	90.414 (15)	Ru1^xxiv^—La4—La2^iv^	136.53 (4)
La4—La1—La2	107.79 (3)	Ru1^iii^—La4—La2^iv^	40.60 (3)
La3^iii^—La1—La2	153.67 (2)	Ru3^xxv^—La4—La2^iv^	131.435 (17)
Ru1^i^—La1—La1^vi^	108.52 (3)	Ru3^ii^—La4—La2^iv^	102.66 (2)
Ru3^ii^—La1—La1^vi^	51.393 (19)	In1^i^—La4—La2^iv^	49.124 (14)
Ru4—La1—La1^vi^	117.89 (3)	In1^xxv^—La4—La2^iv^	91.16 (3)
In2—La1—La1^vi^	54.924 (10)	Ru2^i^—La4—La2^iv^	49.124 (14)
In1^i^—La1—La1^vi^	147.308 (15)	Ru2^xxv^—La4—La2^iv^	91.16 (3)
Ru2^i^—La1—La1^vi^	147.308 (15)	La5—La4—La2^iv^	59.041 (17)
Ru1^iii^—La1—La1^vi^	105.49 (3)	La3^iii^—La4—La2^iv^	82.601 (16)
La2^iv^—La1—La1^vi^	148.189 (14)	La3^xvii^—La4—La2^iv^	151.947 (19)
La1^v^—La1—La1^vi^	90.0	La1—La4—La2^iv^	52.689 (13)
La4—La1—La1^vi^	103.13 (2)	La1^v^—La4—La2^iv^	96.726 (16)
La3^iii^—La1—La1^vi^	59.766 (9)	La1^xxvi^—La4—La2^iv^	96.692 (16)
La2—La1—La1^vi^	144.697 (13)	La1^xxvii^—La4—La2^iv^	147.12 (2)
Ru1^i^—La1—La1^iv^	145.48 (3)	La2^xxviii^—La4—La2^iv^	118.08 (3)
Ru3^ii^—La1—La1^iv^	112.90 (3)	La2^xxv^—La4—La2^iv^	95.95 (3)
Ru4—La1—La1^iv^	51.321 (13)	Ru1^xxiv^—La4—La2^i^	136.53 (4)
In2—La1—La1^iv^	54.641 (5)	Ru1^iii^—La4—La2^i^	40.60 (3)
In1^i^—La1—La1^iv^	116.06 (2)	Ru3^xxv^—La4—La2i	102.66 (2)
Ru2^i^—La1—La1^iv^	116.06 (2)	Ru3i—La4—La2i	131.435 (17)
Ru1^iii^—La1—La1^iv^	47.96 (3)	In1i—La4—La2i	91.16 (3)
La2^iv^—La1—La1^iv^	60.927 (16)	In1i—La4—La2i	49.124 (14)
La1^v^—La1—La1^iv^	159.439 (14)	Ru2i—La4—La2i	91.16 (3)
La4—La1—La1^iv^	99.18 (2)	Ru2i—La4—La2i	49.124 (14)
La3^iii^—La1—La1^iv^	61.58 (3)	La5—La4—La2i	59.041 (17)
La2—La1—La1^iv^	101.314 (14)	La3i—La4—La2i	82.601 (16)
La1^vi^—La1—La1^iv^	90.0	La3i—La4—La2i	151.947 (19)
Ru1^i^—La1—La1^vii^	62.01 (3)	La1—La4—La2i	96.692 (16)
Ru3^ii^—La1—La1^vii^	132.66 (4)	La1i—La4—La2i	147.12 (2)
Ru4—La1—La1^vii^	51.321 (13)	La1i—La4—La2i	52.689 (13)
In2—La1—La1^vii^	54.641 (5)	La1i—La4—La2i	96.726 (16)
In1^i^—La1—La1^vii^	108.31 (3)	La2i—La4—La2i	95.95 (3)
Ru2^i^—La1—La1^vii^	108.31 (3)	La2i—La4—La2i	118.08 (3)
Ru1^iii^—La1—La1^vii^	133.87 (3)	La2i—La4—La2i	50.73 (2)
La2^iv^—La1—La1^vii^	101.787 (14)	In1i—La5—Ru2i	0.00 (5)
La1^v^—La1—La1^vii^	110.561 (14)	In1i—La5—In1i	116.87 (4)
La4—La1—La1^vii^	163.89 (2)	Ru2i—La5—In1i	116.87 (4)
La3^iii^—La1—La1^vii^	136.140 (19)	In1i—La5—Ru2i	116.87 (4)
La2—La1—La1^vii^	57.072 (15)	Ru2i—La5—Ru2i	116.87 (4)
La1^vi^—La1—La1^vii^	90.0	In1i—La5—Ru2i	0.00 (5)
La1^iv^—La1—La1^vii^	90.0	In1i—La5—In1i	105.90 (2)
Ru1^i^—La1—La3^ii^	48.24 (4)	Ru2i—La5—In1i	105.90 (2)
Ru3^ii^—La1—La3^ii^	77.10 (4)	In1i—La5—In1i	105.90 (2)
Ru4—La1—La3^ii^	109.84 (2)	Ru2i—La5—In1i	105.90 (2)
In2—La1—La3^ii^	79.748 (13)	In1i—La5—In1i	105.90 (2)
In1^i^—La1—La3^ii^	105.53 (2)	Ru2i—La5—In1i	105.90 (2)
Ru2^i^—La1—La3^ii^	105.53 (2)	In1i—La5—In1i	105.90 (2)
Ru1^iii^—La1—La3^ii^	163.83 (3)	Ru2i—La5—In1i	105.90 (2)
La2^iv^—La1—La3^ii^	149.48 (2)	In1i—La5—In1i	116.87 (4)
La1^v^—La1—La3^ii^	61.869 (12)	In1i—La5—Ru2i	105.90 (2)
La4—La1—La3^ii^	120.05 (2)	Ru2i—La5—Ru2i	105.90 (2)
La3^iii^—La1—La3^ii^	117.600 (15)	In1i—La5—Ru2i	105.90 (2)
La2—La1—La3^ii^	88.72 (2)	Ru2i—La5—Ru2i	105.90 (2)
La1^vi^—La1—La3^ii^	60.766 (12)	In1i—La5—Ru2i	0.00 (5)
La1^iv^—La1—La3^ii^	134.374 (13)	In1i—La5—Ru2i	116.87 (4)
La1^vii^—La1—La3^ii^	58.54 (2)	In1i—La5—Ru2i	105.90 (2)
Ru1^i^—La2—Ru5	153.94 (4)	Ru2i—La5—Ru2i	105.90 (2)
Ru1^i^—La2—Ru4	96.58 (4)	In1i—La5—Ru2i	105.90 (2)
Ru5—La2—Ru4	61.66 (3)	Ru2i—La5—Ru2i	105.90 (2)
Ru1^i^—La2—In1^i^	66.35 (5)	In1i—La5—Ru2i	116.87 (4)
Ru5—La2—In1^i^	100.77 (3)	In1i—La5—Ru2i	0.00 (5)
Ru4—La2—In1^i^	98.14 (2)	Ru2i—La5—Ru2i	116.87 (4)
Ru1^i^—La2—Ru2^i^	66.35 (5)	In1i—La5—La4i	58.44 (2)
Ru5—La2—Ru2^i^	100.77 (3)	Ru2i—La5—La4i	58.44 (2)
Ru4—La2—Ru2^i^	98.14 (2)	In1i—La5—La4i	58.44 (2)
In1^i^—La2—Ru2^i^	0.00 (5)	Ru2i—La5—La4i	58.44 (2)
Ru1^i^—La2—In1^viii^	97.34 (5)	In1i—La5—La4i	121.56 (2)
Ru5—La2—In1^viii^	99.26 (3)	In1i—La5—La4i	121.56 (2)
Ru4—La2—In1^viii^	96.72 (3)	Ru2i—La5—La4i	121.56 (2)
In1^i^—La2—In1^viii^	159.05 (3)	Ru2i—La5—La4i	121.56 (2)
Ru2^i^—La2—In1^viii^	159.05 (3)	In1i—La5—La4	121.56 (2)
Ru1^i^—La2—Ru2^viii^	97.34 (5)	Ru2i—La5—La4	121.56 (2)
Ru5—La2—Ru2^viii^	99.26 (3)	In1i—La5—La4	121.56 (2)
Ru4—La2—Ru2^viii^	96.72 (3)	Ru2i—La5—La4	121.56 (2)
In1^i^—La2—Ru2^viii^	159.05 (3)	In1i—La5—La4	58.44 (2)
Ru2^i^—La2—Ru2^viii^	159.05 (3)	In1i—La5—La4	58.44 (2)
In1^viii^—La2—Ru2^viii^	0.00 (5)	Ru2i—La5—La4	58.44 (2)
Ru1^i^—La2—La2^ix^	152.02 (3)	Ru2i—La5—La4	58.44 (2)
Ru5—La2—La2^ix^	54.020 (13)	La4i—La5—La4	180.0
Ru4—La2—La2^ix^	106.31 (3)	In1i—La5—La2i	151.770 (11)
In1^i^—La2—La2^ix^	124.21 (4)	Ru2i—La5—La2i	151.770 (11)
Ru2^i^—La2—La2^ix^	124.21 (4)	In1i—La5—La2i	60.91 (2)
In1^viii^—La2—La2^ix^	64.66 (3)	Ru2i—La5—La2i	60.91 (2)
Ru2^viii^—La2—La2^ix^	64.66 (3)	In1i—La5—La2i	101.346 (16)
Ru1^i^—La2—La1^vii^	66.18 (3)	In1i—La5—La2i	53.724 (10)
Ru5—La2—La1^vii^	105.49 (2)	Ru2i—La5—La2i	101.346 (16)
Ru4—La2—La1^vii^	53.12 (3)	Ru2i—La5—La2i	53.724 (10)
In1^i^—La2—La1^vii^	119.10 (3)	La4i—La5—La2i	112.164 (9)
Ru2^i^—La2—La1^vii^	119.10 (3)	La4—La5—La2i	67.836 (9)
In1^viii^—La2—La1^vii^	60.38 (3)	In1i—La5—La2i	151.770 (11)
Ru2^viii^—La2—La1^vii^	60.38 (3)	Ru2i—La5—La2i	151.770 (11)
La2^ix^—La2—La1^vii^	115.72 (3)	In1i—La5—La2i	60.91 (2)
Ru1^i^—La2—La2^x^	128.22 (4)	Ru2i—La5—La2i	60.91 (2)
Ru5—La2—La2^x^	52.682 (13)	In1i—La5—La2i	53.724 (10)
Ru4—La2—La2^x^	103.66 (3)	In1i—La5—La2i	101.346 (16)
In1^i^—La2—La2^x^	63.93 (3)	Ru2i—La5—La2i	53.724 (10)
Ru2^i^—La2—La2^x^	63.93 (3)	Ru2i—La5—La2i	101.346 (16)
In1^viii^—La2—La2^x^	126.15 (4)	La4i—La5—La2i	112.164 (9)
Ru2^viii^—La2—La2^x^	126.15 (4)	La4—La5—La2i	67.836 (9)
La2^ix^—La2—La2^x^	61.90 (2)	La2i—La5—La2i	55.12 (2)
La1^vii^—La2—La2^x^	156.27 (2)	In1i—La5—La2i	53.724 (10)
Ru1^i^—La2—In1^xi^	108.17 (3)	Ru2i—La5—La2i	53.724 (10)
Ru5—La2—In1^xi^	91.53 (2)	In1i—La5—La2i	101.346 (16)
Ru4—La2—In1^xi^	152.93 (4)	Ru2i—La5—La2i	101.346 (16)
In1^i^—La2—In1^xi^	82.45 (3)	In1i—La5—La2i	151.770 (11)
Ru2^i^—La2—In1^xi^	82.45 (3)	In1i—La5—La2i	60.91 (2)
In1^viii^—La2—In1^xi^	90.96 (4)	Ru2i—La5—La2i	151.770 (11)
Ru2^viii^—La2—In1^xi^	90.96 (4)	Ru2i—La5—La2i	60.91 (2)
La2^ix^—La2—In1^xi^	54.223 (18)	La4i—La5—La2i	67.836 (9)
La1^vii^—La2—In1^xi^	148.25 (3)	La4—La5—La2i	112.164 (9)
La2^x^—La2—In1^xi^	52.17 (2)	La2i—La5—La2i	98.183 (7)
Ru1^i^—La2—Ru2^xi^	108.17 (3)	La2i—La5—La2i	152.21 (2)
Ru5—La2—Ru2^xi^	91.53 (2)	In1i—La5—La2i	53.724 (10)
Ru4—La2—Ru2^xi^	152.93 (4)	Ru2i—La5—La2i	53.724 (10)
In1^i^—La2—Ru2^xi^	82.45 (3)	In1i—La5—La2i	101.346 (16)
Ru2^i^—La2—Ru2^xi^	82.45 (3)	Ru2i—La5—La2i	101.346 (16)
In1^viii^—La2—Ru2^xi^	90.96 (4)	In1i—La5—La2i	60.91 (2)
Ru2^viii^—La2—Ru2^xi^	90.96 (4)	In1i—La5—La2i	151.770 (11)
La2^ix^—La2—Ru2^xi^	54.223 (18)	Ru2i—La5—La2i	60.91 (2)
La1^vii^—La2—Ru2^xi^	148.25 (3)	Ru2i—La5—La2i	151.770 (11)
La2^x^—La2—Ru2^xi^	52.17 (2)	La4i—La5—La2i	67.836 (9)
In1^xi^—La2—Ru2^xi^	0.00 (2)	La4—La5—La2i	112.164 (9)
Ru1^i^—La2—La2^v^	48.86 (3)	La2i—La5—La2i	152.21 (2)
Ru5—La2—La2^v^	142.682 (13)	La2i—La5—La2i	98.183 (7)
Ru4—La2—La2^v^	140.98 (2)	La2i—La5—La2i	106.69 (2)
In1^i^—La2—La2^v^	55.251 (18)	In1i—La5—La2i	60.91 (2)
Ru2^i^—La2—La2^v^	55.251 (18)	Ru2i—La5—La2i	60.91 (2)
In1^viii^—La2—La2^v^	104.32 (2)	In1i—La5—La2i	151.770 (11)
Ru2^viii^—La2—La2^v^	104.32 (2)	Ru2i—La5—La2i	151.770 (11)
La2^ix^—La2—La2^v^	112.316 (14)	In1i—La5—La2i	101.346 (16)
La1^vii^—La2—La2^v^	111.218 (15)	In1i—La5—La2i	53.724 (10)
La2^x^—La2—La2^v^	90.0	Ru2i—La5—La2i	101.346 (16)
In1^xi^—La2—La2^v^	59.923 (14)	Ru2i—La5—La2i	53.724 (10)
Ru2^xi^—La2—La2^v^	59.923 (14)	La4i—La5—La2i	112.164 (9)
Ru1^i^—La2—La2^vii^	127.67 (3)	La4—La5—La2i	67.836 (9)
Ru5—La2—La2^vii^	52.110 (5)	La2i—La5—La2i	106.69 (2)
Ru4—La2—La2^vii^	53.130 (14)	La2i—La5—La2i	135.671 (18)
In1^i^—La2—La2^vii^	146.01 (3)	La2i—La5—La2i	53.130 (19)
Ru2^i^—La2—La2^vii^	146.01 (3)	La2i—La5—La2i	98.183 (7)
In1^viii^—La2—La2^vii^	54.40 (3)	In1i—La5—La2i	60.91 (2)
Ru2^viii^—La2—La2^vii^	54.40 (3)	Ru2i—La5—La2i	60.91 (2)
La2^ix^—La2—La2^vii^	60.55 (2)	In1i—La5—La2i	151.770 (11)
La1^vii^—La2—La2^vii^	61.549 (16)	Ru2i—La5—La2i	151.770 (11)
La2^x^—La2—La2^vii^	102.075 (5)	In1i—La5—La2i	53.724 (10)
In1^xi^—La2—La2^vii^	114.454 (19)	In1i—La5—La2i	101.346 (16)
Ru2^xi^—La2—La2^vii^	114.454 (19)	Ru2i—La5—La2i	53.724 (10)
La2^v^—La2—La2^vii^	158.701 (16)	Ru2i—La5—La2i	101.346 (16)
Ru1^i^—La2—La2^iv^	104.12 (4)	La4i—La5—La2i	112.164 (9)
Ru5—La2—La2^iv^	52.110 (5)	La4—La5—La2i	67.836 (9)
Ru4—La2—La2^iv^	53.130 (14)	La2i—La5—La2i	135.671 (18)
In1^i^—La2—La2^iv^	56.11 (3)	La2i—La5—La2i	106.69 (2)
Ru2^i^—La2—La2^iv^	56.11 (3)	La2i—La5—La2i	98.183 (7)
In1^viii^—La2—La2^iv^	144.30 (3)	La2i—La5—La2i	53.130 (19)
Ru2^viii^—La2—La2^iv^	144.30 (3)	La2i—La5—La2i	55.12 (2)
La2^ix^—La2—La2^iv^	102.465 (5)	In1i—La5—La2i	101.346 (16)
La1^vii^—La2—La2^iv^	103.037 (14)	Ru2i—La5—La2i	101.346 (16)
La2^x^—La2—La2^iv^	57.55 (2)	In1i—La5—La2i	53.724 (10)
In1^xi^—La2—La2^iv^	108.54 (3)	Ru2i—La5—La2i	53.724 (10)
Ru2^xi^—La2—La2^iv^	108.54 (3)	In1i—La5—La2i	151.770 (11)
La2^v^—La2—La2^iv^	111.299 (16)	In1i—La5—La2i	60.91 (2)
La2^vii^—La2—La2^iv^	90.0	Ru2i—La5—La2i	151.770 (11)
Ru1^i^—La2—La1	51.96 (3)	Ru2i—La5—La2i	60.91 (2)
Ru5—La2—La1	102.03 (2)	La4i—La5—La2i	67.836 (9)
Ru4—La2—La1	51.44 (2)	La4—La5—La2i	112.164 (9)
In1^i^—La2—La1	59.24 (3)	La2i—La5—La2i	53.130 (19)
Ru2^i^—La2—La1	59.24 (3)	La2i—La5—La2i	98.183 (7)
In1^viii^—La2—La1	121.93 (3)	La2i—La5—La2i	55.12 (2)
Ru2^viii^—La2—La1	121.93 (3)	La2i—La5—La2i	135.671 (18)
La2^ix^—La2—La1	155.591 (17)	La2i—La5—La2i	98.183 (7)
La1^vii^—La2—La1	62.001 (19)	La2i—La5—La2i	152.21 (2)
La2^x^—La2—La1	109.54 (3)	In1i—La5—La2i	101.346 (16)
In1^xi^—La2—La1	140.96 (3)	Ru2i—La5—La2i	101.346 (16)
Ru2^xi^—La2—La1	140.96 (3)	In1i—La5—La2i	53.724 (10)
La2^v^—La2—La1	89.586 (15)	Ru2i—La5—La2i	53.724 (10)
La2^vii^—La2—La1	102.512 (13)	In1i—La5—La2i	60.91 (2)
La2^iv^—La2—La1	57.604 (16)	In1i—La5—La2i	151.770 (11)
Ru1^i^—La2—La5^xii^	88.59 (4)	Ru2i—La5—La2i	60.91 (2)
Ru5—La2—La5^xii^	117.455 (19)	Ru2i—La5—La2i	151.770 (11)
Ru4—La2—La5^xii^	146.38 (2)	La4i—La5—La2i	67.836 (9)
In1^i^—La2—La5^xii^	114.20 (2)	La4—La5—La2i	112.164 (9)
Ru2^i^—La2—La5^xii^	114.20 (2)	La2i—La5—La2i	98.183 (7)
In1^viii^—La2—La5^xii^	49.67 (2)	La2i—La5—La2i	53.130 (19)
Ru2^viii^—La2—La5^xii^	49.67 (2)	La2i—La5—La2i	135.671 (18)
La2^ix^—La2—La5^xii^	63.435 (9)	La2i—La5—La2i	55.12 (2)
La1^vii^—La2—La5^xii^	100.404 (19)	La2i—La5—La2i	152.21 (2)
La2^x^—La2—La5^xii^	98.84 (2)	La2i—La5—La2i	98.183 (7)
In1^xi^—La2—La5^xii^	47.88 (2)	La2i—La5—La2i	106.69 (2)
Ru2^xi^—La2—La5^xii^	47.88 (2)	La2i—Ru1—La2i	82.28 (5)
La2^v^—La2—La5^xii^	62.442 (10)	La2i—Ru1—La3	137.34 (3)
La2^vii^—La2—La5^xii^	98.03 (2)	La2i—Ru1—La3	137.34 (3)
La2^iv^—La2—La5^xii^	156.280 (13)	La2i—Ru1—La1i	129.38 (7)
La1—La2—La5^xii^	140.19 (2)	La2i—Ru1—La1i	80.57 (3)
Ru1^i^—La2—La4^xiii^	47.69 (4)	La3—Ru1—La1i	81.52 (4)
Ru5—La2—La4^xiii^	148.76 (2)	La2i—Ru1—La1i	80.57 (3)
Ru4—La2—La4^xiii^	108.30 (3)	La2i—Ru1—La1i	129.38 (7)
In1^i^—La2—La4^xiii^	110.16 (3)	La3—Ru1—La1i	81.52 (4)
Ru2^i^—La2—La4^xiii^	110.16 (3)	La1i—Ru1—La1i	74.69 (5)
In1^viii^—La2—La4^xiii^	50.68 (2)	La2i—Ru1—La4i	91.71 (5)
Ru2^viii^—La2—La4^xiii^	50.68 (2)	La2i—Ru1—La4i	91.71 (5)
La2^ix^—La2—La4^xiii^	108.33 (2)	La3—Ru1—La4i	75.27 (6)
La1^vii^—La2—La4^xiii^	55.473 (19)	La1i—Ru1—La4i	135.90 (4)
La2^x^—La2—La4^xiii^	148.03 (2)	La1i—Ru1—La4i	135.90 (4)
In1^xi^—La2—La4^xiii^	96.65 (3)	La2i—Ru1—La1i	67.76 (2)
Ru2^xi^—La2—La4^xiii^	96.65 (3)	La2i—Ru1—La1i	141.71 (5)
La2^v^—La2—La4^xiii^	64.635 (10)	La3—Ru1—La1i	69.77 (3)
La2^vii^—La2—La4^xiii^	97.43 (2)	La1i—Ru1—La1i	136.97 (5)
La2^iv^—La2—La4^xiii^	147.966 (18)	La1i—Ru1—La1i	70.03 (2)
La1—La2—La4^xiii^	90.36 (2)	La4i—Ru1—La1i	66.92 (3)
La5^xii^—La2—La4^xiii^	53.123 (17)	La2i—Ru1—La1i	141.71 (5)
Ru1^vi^—La3—Ru1	154.28 (9)	La2i—Ru1—La1i	67.76 (2)
Ru1^vi^—La3—Ru3^xiv^	100.76 (4)	La3—Ru1—La1i	69.77 (3)
Ru1—La3—Ru3^xiv^	100.76 (4)	La1i—Ru1—La1i	70.03 (2)
Ru1^vi^—La3—Ru3^xv^	100.76 (4)	La1i—Ru1—La1i	136.97 (5)
Ru1—La3—Ru3^xv^	100.76 (4)	La4i—Ru1—La1i	66.92 (3)
Ru3^xiv^—La3—Ru3^xv^	65.99 (7)	La1i—Ru1—La1i	124.26 (6)
Ru1^vi^—La3—La4^xvi^	149.96 (5)	La5i—Ru2—La2i	132.69 (3)
Ru1—La3—La4^xvi^	55.75 (4)	La5i—Ru2—La2i	132.69 (3)
Ru3^xiv^—La3—La4^xvi^	55.19 (2)	La2i—Ru2—La2i	69.50 (4)
Ru3^xv^—La3—La4^xvi^	55.19 (2)	La5i—Ru2—La2i	76.61 (3)
Ru1^vi^—La3—La4^xvii^	55.75 (4)	La2i—Ru2—La2i	69.49 (3)
Ru1—La3—La4^xvii^	149.96 (5)	La2i—Ru2—La2i	138.87 (3)
Ru3^xiv^—La3—La4^xvii^	55.19 (2)	La5i—Ru2—La2i	76.61 (3)
Ru3^xv^—La3—La4^xvii^	55.19 (2)	La2i—Ru2—La2i	138.87 (3)
La4^xvi^—La3—La4^xvii^	94.21 (4)	La2i—Ru2—La2i	69.49 (3)
Ru1^vi^—La3—La1^xvi^	122.257 (9)	La2i—Ru2—La2i	150.98 (5)
Ru1—La3—La1^xvi^	63.380 (15)	La5i—Ru2—La4i	69.62 (3)
Ru3^xiv^—La3—La1^xvi^	51.68 (3)	La2i—Ru2—La4i	132.16 (3)
Ru3^xv^—La3—La1^xvi^	107.30 (4)	La2i—Ru2—La4i	132.16 (3)
La4^xvi^—La3—La1^xvi^	60.009 (14)	La2i—Ru2—La4i	80.19 (2)
La4^xvii^—La3—La1^xvi^	103.76 (3)	La2i—Ru2—La4i	80.19 (2)
Ru1^vi^—La3—La1^xviii^	63.380 (15)	La5i—Ru2—La1i	124.03 (3)
Ru1—La3—La1^xviii^	122.257 (9)	La2i—Ru2—La1i	67.79 (3)
Ru3^xiv^—La3—La1^xviii^	107.30 (4)	La2i—Ru2—La1i	102.52 (4)
Ru3^xv^—La3—La1^xviii^	51.68 (3)	La2i—Ru2—La1i	64.082 (19)
La4^xvi^—La3—La1^xviii^	103.76 (3)	La2i—Ru2—La1i	124.96 (4)
La4^xvii^—La3—La1^xviii^	60.009 (14)	La4i—Ru2—La1i	65.88 (3)
La1^xvi^—La3—La1^xviii^	157.81 (4)	La5i—Ru2—La1i	124.03 (3)
Ru1^vi^—La3—La1^xix^	63.380 (15)	La2i—Ru2—La1i	102.52 (4)
Ru1—La3—La1^xix^	122.257 (9)	La2i—Ru2—La1i	67.79 (3)
Ru3^xiv^—La3—La1^xix^	51.68 (3)	La2i—Ru2—La1i	124.96 (4)
Ru3^xv^—La3—La1^xix^	107.30 (4)	La2i—Ru2—La1i	64.082 (19)
La4^xvi^—La3—La1^xix^	103.76 (3)	La4i—Ru2—La1i	65.88 (3)
La4^xvii^—La3—La1^xix^	60.009 (14)	La1i—Ru2—La1i	62.89 (3)
La1^xvi^—La3—La1^xix^	60.467 (19)	La5i—Ru2—La2i	71.20 (3)
La1^xviii^—La3—La1^xix^	114.76 (2)	La2i—Ru2—La2i	63.90 (3)
Ru1^vi^—La3—La1^xx^	122.257 (9)	La2i—Ru2—La2i	97.55 (3)
Ru1—La3—La1^xx^	63.380 (15)	La2i—Ru2—La2i	61.12 (2)
Ru3^xiv^—La3—La1^xx^	107.30 (4)	La2i—Ru2—La2i	119.17 (4)
Ru3^xv^—La3—La1^xx^	51.68 (3)	La4i—Ru2—La2i	129.84 (4)
La4^xvi^—La3—La1^xx^	60.009 (14)	La1i—Ru2—La2i	115.849 (17)
La4^xvii^—La3—La1^xx^	103.76 (3)	La1i—Ru2—La2i	163.52 (4)
La1^xvi^—La3—La1^xx^	114.76 (2)	La5i—Ru2—La2i	71.20 (3)
La1^xviii^—La3—La1^xx^	60.467 (19)	La2i—Ru2—La2i	97.55 (3)
La1^xix^—La3—La1^xx^	157.81 (4)	La2i—Ru2—La2i	63.90 (3)
Ru1^vi^—La3—La1^xiv^	50.24 (3)	La2i—Ru2—La2i	119.17 (4)
Ru1—La3—La1^xiv^	108.22 (4)	La2i—Ru2—La2i	61.12 (2)
Ru3^xiv^—La3—La1^xiv^	150.765 (12)	La4i—Ru2—La2i	129.84 (4)
Ru3^xv^—La3—La1^xiv^	111.05 (3)	La1i—Ru2—La2i	163.52 (4)
La4^xvi^—La3—La1^xiv^	149.047 (11)	La1i—Ru2—La2i	115.849 (17)
La4^xvii^—La3—La1^xiv^	98.166 (15)	La2i—Ru2—La2i	60.15 (3)
La1^xvi^—La3—La1^xiv^	141.66 (3)	La1i—Ru3—La1i	77.21 (4)
La1^xviii^—La3—La1^xiv^	59.886 (19)	La1i—Ru3—La1i	120.29 (7)
La1^xix^—La3—La1^xiv^	107.008 (19)	La1i—Ru3—La1i	74.08 (4)
La1^xx^—La3—La1^xiv^	89.43 (2)	La1i—Ru3—La1i	74.08 (4)
Ru1^vi^—La3—La1^xxi^	50.24 (3)	La1i—Ru3—La1i	120.29 (7)
Ru1—La3—La1^xxi^	108.22 (4)	La1i—Ru3—La1i	77.21 (4)
Ru3^xiv^—La3—La1^xxi^	111.04 (3)	La1i—Ru3—La3i	76.459 (16)
Ru3^xv^—La3—La1^xxi^	150.765 (12)	La1i—Ru3—La3i	76.459 (16)
La4^xvi^—La3—La1^xxi^	149.047 (11)	La1i—Ru3—La3i	140.924 (12)
La4^xvii^—La3—La1^xxi^	98.166 (15)	La1i—Ru3—La3i	140.924 (12)
La1^xvi^—La3—La1^xxi^	89.43 (2)	La1i—Ru3—La3i	140.924 (12)
La1^xviii^—La3—La1^xxi^	107.008 (19)	La1i—Ru3—La3i	140.924 (12)
La1^xix^—La3—La1^xxi^	59.886 (19)	La1i—Ru3—La3i	76.459 (16)
La1^xx^—La3—La1^xxi^	141.66 (3)	La1i—Ru3—La3i	76.459 (16)
La1^xiv^—La3—La1^xxi^	56.26 (2)	La3i—Ru3—La3i	114.01 (7)
Ru1^vi^—La3—La1^xxii^	108.22 (4)	La1i—Ru3—La4i	73.911 (15)
Ru1—La3—La1^xxii^	50.24 (3)	La1i—Ru3—La4i	142.318 (12)
Ru3^xiv^—La3—La1^xxii^	150.765 (12)	La1i—Ru3—La4i	142.317 (12)
Ru3^xv^—La3—La1^xxii^	111.05 (3)	La1i—Ru3—La4i	73.911 (15)
La4^xvi^—La3—La1^xxii^	98.166 (15)	La3i—Ru3—La4i	73.66 (3)
La4^xvii^—La3—La1^xxii^	149.047 (11)	La3i—Ru3—La4i	73.66 (3)
La1^xvi^—La3—La1^xxii^	107.008 (19)	La1i—Ru3—La4i	142.317 (12)
La1^xviii^—La3—La1^xxii^	89.43 (2)	La1i—Ru3—La4i	73.911 (15)
La1^xix^—La3—La1^xxii^	141.66 (3)	La1i—Ru3—La4i	73.911 (15)
La1^xx^—La3—La1^xxii^	59.886 (19)	La1i—Ru3—La4i	142.318 (12)
La1^xiv^—La3—La1^xxii^	58.47 (2)	La3i—Ru3—La4i	73.66 (3)
La1^xxi^—La3—La1^xxii^	85.50 (3)	La3i—Ru3—La4i	73.66 (3)
Ru1^vi^—La3—La1^xxiii^	108.22 (4)	La4i—Ru3—La4i	117.80 (7)
Ru1—La3—La1^xxiii^	50.24 (3)	La1i—Ru4—La1i	77.36 (3)
Ru3^xiv^—La3—La1^xxiii^	111.04 (3)	La1i—Ru4—La1	124.21 (6)
Ru3^xv^—La3—La1^xxiii^	150.765 (12)	La1i—Ru4—La1	77.36 (3)
La4^xvi^—La3—La1^xxiii^	98.166 (15)	La1i—Ru4—La1i	77.36 (3)
La4^xvii^—La3—La1^xxiii^	149.047 (11)	La1i—Ru4—La1i	124.21 (6)
La1^xvi^—La3—La1^xxiii^	59.886 (19)	La1—Ru4—La1i	77.36 (3)
La1^xviii^—La3—La1^xxiii^	141.66 (3)	La1i—Ru4—La2	138.418 (17)
La1^xix^—La3—La1^xxiii^	89.43 (2)	La1i—Ru4—La2	143.716 (18)
La1^xx^—La3—La1^xxiii^	107.008 (19)	La1—Ru4—La2	75.350 (16)
La1^xiv^—La3—La1^xxiii^	85.50 (3)	La1i—Ru4—La2	71.881 (16)
La1^xxi^—La3—La1^xxiii^	58.47 (2)	La1i—Ru4—La2i	143.716 (18)
La1^xxii^—La3—La1^xxiii^	56.26 (2)	La1i—Ru4—La2i	75.350 (16)
Ru1^xxiv^—La4—Ru1^iii^	176.27 (8)	La1—Ru4—La2i	71.881 (16)
Ru1^xxiv^—La4—Ru3^xxv^	91.60 (3)	La1i—Ru4—La2i	138.418 (17)
Ru1^iii^—La4—Ru3^xxv^	91.60 (3)	La2—Ru4—La2i	73.74 (3)
Ru1^xxiv^—La4—Ru3^ii^	91.60 (3)	La1i—Ru4—La2i	75.350 (16)
Ru1^iii^—La4—Ru3^ii^	91.60 (3)	La1i—Ru4—La2i	71.881 (16)
Ru3^xxv^—La4—Ru3^ii^	62.20 (7)	La1—Ru4—La2i	138.418 (17)
Ru1^xxiv^—La4—In1^i^	88.85 (2)	La1i—Ru4—La2i	143.716 (18)
Ru1^iii^—La4—In1^i^	88.85 (2)	La2—Ru4—La2i	116.11 (6)
Ru3^xxv^—La4—In1^i^	159.16 (5)	La2i—Ru4—La2i	73.74 (3)
Ru3^ii^—La4—In1^i^	96.95 (4)	La1i—Ru4—La2i	71.881 (16)
Ru1^xxiv^—La4—In1^xxv^	88.85 (2)	La1i—Ru4—La2i	138.418 (17)
Ru1^iii^—La4—In1^xxv^	88.85 (2)	La1—Ru4—La2i	143.716 (18)
Ru3^xxv^—La4—In1^xxv^	96.95 (4)	La1i—Ru4—La2i	75.350 (16)
Ru3^ii^—La4—In1^xxv^	159.16 (5)	La2—Ru4—La2i	73.74 (3)
In1^i^—La4—In1^xxv^	103.89 (6)	La2i—Ru4—La2i	116.11 (6)
Ru1^xxiv^—La4—Ru2^i^	88.85 (2)	La2i—Ru4—La2i	73.74 (3)
Ru1^iii^—La4—Ru2^i^	88.85 (2)	La2i—Ru5—La2i	139.13 (3)
Ru3^xxv^—La4—Ru2^i^	159.16 (5)	La2i—Ru5—La2i	143.22 (3)
Ru3^ii^—La4—Ru2^i^	96.95 (4)	La2i—Ru5—La2i	75.781 (11)
In1^i^—La4—Ru2^i^	0.00 (4)	La2i—Ru5—La2i	75.781 (11)
In1^xxv^—La4—Ru2^i^	103.89 (6)	La2i—Ru5—La2i	143.22 (3)
Ru1^xxiv^—La4—Ru2^xxv^	88.85 (2)	La2i—Ru5—La2i	74.64 (3)
Ru1^iii^—La4—Ru2^xxv^	88.85 (2)	La2i—Ru5—La2i	120.58 (2)
Ru3^xxv^—La4—Ru2^xxv^	96.95 (4)	La2i—Ru5—La2i	74.64 (3)
Ru3^ii^—La4—Ru2^xxv^	159.16 (5)	La2i—Ru5—La2i	71.96 (3)
In1^i^—La4—Ru2^xxv^	103.89 (6)	La2i—Ru5—La2i	75.781 (11)
In1^xxv^—La4—Ru2^xxv^	0.00 (4)	La2i—Ru5—La2	74.64 (3)
Ru2^i^—La4—Ru2^xxv^	103.89 (6)	La2i—Ru5—La2	120.58 (2)
Ru1^xxiv^—La4—La5	88.13 (4)	La2i—Ru5—La2	75.781 (11)
Ru1^iii^—La4—La5	88.13 (4)	La2i—Ru5—La2	71.96 (3)
Ru3^xxv^—La4—La5	148.90 (3)	La2i—Ru5—La2	139.13 (3)
Ru3^ii^—La4—La5	148.90 (3)	La2i—Ru5—La2i	75.781 (11)
In1^i^—La4—La5	51.94 (3)	La2i—Ru5—La2i	71.96 (3)
In1^xxv^—La4—La5	51.94 (3)	La2i—Ru5—La2i	139.13 (3)
Ru2^i^—La4—La5	51.94 (3)	La2i—Ru5—La2i	120.58 (2)
Ru2^xxv^—La4—La5	51.94 (3)	La2i—Ru5—La2i	75.781 (11)
Ru1^xxiv^—La4—La3^iii^	134.76 (5)	La2—Ru5—La2i	143.22 (3)
Ru1^iii^—La4—La3^iii^	48.97 (3)	La2i—Ru5—La2i	71.96 (3)
Ru3^xxv^—La4—La3^iii^	51.15 (3)	La2i—Ru5—La2i	75.781 (11)
Ru3^ii^—La4—La3^iii^	51.15 (3)	La2i—Ru5—La2i	120.58 (2)
In1^i^—La4—La3^iii^	116.846 (17)	La2i—Ru5—La2i	139.13 (3)
In1^xxv^—La4—La3^iii^	116.845 (17)	La2i—Ru5—La2i	143.22 (3)
Ru2^i^—La4—La3^iii^	116.846 (17)	La2—Ru5—La2i	75.781 (11)
Ru2^xxv^—La4—La3^iii^	116.845 (17)	La2i—Ru5—La2i	74.64 (3)
La5—La4—La3^iii^	137.10 (2)	La1—In2—La1i	180.00 (3)
Ru1^xxiv^—La4—La3^xvii^	48.97 (3)	La1—In2—La1i	70.717 (10)
Ru1^iii^—La4—La3^xvii^	134.76 (5)	La1i—In2—La1i	109.283 (10)
Ru3^xxv^—La4—La3^xvii^	51.15 (3)	La1—In2—La1i	109.85 (2)
Ru3^ii^—La4—La3^xvii^	51.15 (3)	La1i—In2—La1i	70.15 (2)
In1^i^—La4—La3^xvii^	116.845 (17)	La1i—In2—La1i	70.717 (10)
In1^xxv^—La4—La3^xvii^	116.845 (17)	La1—In2—La1i	70.717 (10)
Ru2^i^—La4—La3^xvii^	116.845 (17)	La1i—In2—La1i	109.283 (10)
Ru2^xxv^—La4—La3^xvii^	116.845 (17)	La1i—In2—La1i	109.85 (2)
La5—La4—La3^xvii^	137.10 (2)	La1i—In2—La1i	70.717 (10)
La3^iii^—La4—La3^xvii^	85.79 (4)	La1—In2—La1i	109.283 (10)
Ru1^xxiv^—La4—La1	119.600 (14)	La1i—In2—La1i	70.717 (10)
Ru1^iii^—La4—La1	61.370 (13)	La1i—In2—La1i	70.15 (2)
Ru3^xxv^—La4—La1	103.96 (4)	La1i—In2—La1i	109.283 (10)
Ru3^ii^—La4—La1	50.94 (3)	La1i—In2—La1i	180.00 (3)
In1^i^—La4—La1	58.39 (2)	La1—In2—La1i	70.15 (2)
In1^xxv^—La4—La1	143.53 (3)	La1i—In2—La1i	109.85 (2)
Ru2^i^—La4—La1	58.39 (2)	La1i—In2—La1i	109.283 (10)
Ru2^xxv^—La4—La1	143.53 (3)	La1i—In2—La1i	180.00 (2)
La5—La4—La1	103.13 (2)	La1i—In2—La1i	109.283 (10)
La3^iii^—La4—La1	60.151 (15)	La1i—In2—La1i	70.717 (10)
La3^xvii^—La4—La1	99.50 (3)	La1—In2—La1i	109.283 (10)
Ru1^xxiv^—La4—La1^v^	61.370 (13)	La1i—In2—La1i	70.717 (10)
Ru1^iii^—La4—La1^v^	119.600 (14)	La1i—In2—La1i	180.00 (2)
Ru3^xxv^—La4—La1^v^	103.96 (4)	La1i—In2—La1i	109.283 (10)
Ru3^ii^—La4—La1^v^	50.94 (3)	La1i—In2—La1i	70.15 (2)
In1^i^—La4—La1^v^	58.39 (2)	La1i—In2—La1i	109.85 (2)
In1^xxv^—La4—La1^v^	143.53 (3)	La1i—In2—La1i	70.717 (10)
Ru2^i^—La4—La1^v^	58.39 (2)		

Symmetry codes: (i) *x*−1/2, −*y*+1/2, *z*; (ii) *x*−1/2, −*y*+1/2, −*z*; (iii) *x*−1, *y*, *z*; (iv) −*y*, *x*, *z*; (v) −*y*+1/2, −*x*+1/2, *z*; (vi) *x*, *y*, −*z*; (vii) *y*, −*x*, *z*; (viii) −*x*+1, −*y*, *z*; (ix) *x*, −*y*, −*z*+1/2; (x) *y*, *x*, −*z*+1/2; (xi) −*x*+1, *y*, −*z*+1/2; (xii) −*x*+1/2, −*y*+1/2, −*z*+1/2; (xiii) −*x*+1/2, *y*−1/2, *z*; (xiv) *x*+1/2, −*y*+1/2, −*z*; (xv) −*x*+3/2, *y*+1/2, *z*; (xvi) *x*+1, *y*, *z*; (xvii) −*x*+1, −*y*+1, −*z*; (xviii) *y*+1/2, *x*+1/2, −*z*; (xix) *x*+1, *y*, −*z*; (xx) *y*+1/2, *x*+1/2, *z*; (xxi) −*y*+1, *x*, −*z*; (xxii) *x*+1/2, −*y*+1/2, *z*; (xxiii) −*y*+1, *x*, *z*; (xxiv) −*x*+1, −*y*+1, *z*; (xxv) −*x*+1/2, *y*+1/2, *z*; (xxvi) *y*−1/2, *x*+1/2, *z*; (xxvii) −*x*, −*y*+1, *z*; (xxviii) *y*, −*x*+1, *z*; (xxix) *x*−1/2, *y*+1/2, −*z*+1/2; (xxx) *x*, −*y*+1, −*z*+1/2; (xxxi) −*y*, −*x*+1, −*z*+1/2; (xxxii) *y*+1/2, −*x*+1/2, −*z*+1/2; (xxxiii) −*x*+3/2, *y*−1/2, *z*; (xxxiv) −*x*, −*y*, *z*; (xxxv) −*y*, −*x*, −*z*+1/2; (xxxvi) −*x*, *y*, −*z*+1/2; (xxxvii) −*x*, −*y*, −*z*; (xxxviii) −*y*, *x*, −*z*; (xxxix) *y*, −*x*, −*z*.
